# 3D-printed cranial helmet therapy for the treatment of deformational plagiocephaly

**DOI:** 10.3389/fped.2025.1638581

**Published:** 2025-07-31

**Authors:** Huthaifa Atallah, Rabee Naeem, Raghad Albeetar, Titeana Qufabz, Mahmoud AlFatafta, Amneh Alshawabka, Anas S. Said, Anthony McGarry, Evelin Derkács, Dorottya Varga, Bálint Molics

**Affiliations:** ^1^ Prosthetics and Orthotics Department, School of Rehabilitation Sciences, The University of Jordan, Amman, Jordan; ^2^ Digital Fabrication Division, Revolutionary Technologies for Medical Solutions, Amman, Jordan; ^3^ Neurosurgery Department, Al Basheer Hospital, Amman, Jordan; ^4^ Biomedical Engineering Department, University of Strathclyde, Glasgow, United Kingdom; ^5^ Doctoral School of Health Sciences, Faculty of Health Sciences, University of Pécs, Pécs, Hungary; ^6^ Department of Sport Physiotherapy, Faculty of Health Sciences, University of Pécs, Pécs, Hungary

**Keywords:** 3D-printed helmet, deformational plagiocephaly, CVAI, cranial deformity, cranial helmet therapy

## Abstract

**Introduction:**

Deformational Plagiocephaly (DP) is the most common cranial deformity in infants. It may be treated using molding cranial helmet therapy (CHT) or active counter-positioning (ACP). Molding CHT has proven to be highly effective, especially in moderate to severe cases. Although many studies have explored this topic, few have investigated the use of 3D-printed CHT. This method may offer greater accuracy and convenience in measurement compared to traditional helmet types. Furthermore, no studies on this subject have been conducted in the Middle East.

**Study design:**

A retrospective study design.

**Methods:**

Electronic medical records from the only medical center fitting infants with 3D-printed CHT were reviewed. Infants diagnosed with DP who were fitted with and completed treatment using 3D-printed CHT were included. Infants who received 3D-printed CHT for other cranial deformities were excluded. Descriptive statistics (mean ± SD) were used to present results related to Cranial Vault Asymmetry Index (CVAI) and participants' characteristics. A Linear Mixed Model was used to assess changes in CVAI over time, accounting for age, gender, and treatment duration. Model assumptions were tested, and findings were validated using a Wilcoxon signed-rank test.

**Results:**

Records of eleven infants diagnosed with DP were included, eight boys and three girls. A significant reduction in CVAI was reported in all cases. No significant correlation was found between CVAI improvement and gender, age, or treatment duration.

**Conclusion:**

Following treatment with customized 3D-printed CHT, infants in the study demonstrated significant improvement in the CVAI. The helmets effectively guided cranial growth toward the flattened area, aiding in the correction of the deformity. Although 3D-printed CHT showed results comparable to traditional molding CHT, it offers potential advantages such as increased measurement accuracy through 3D scanning, easier monitoring of progress, and reduced cost and time associated with fabrication through 3D printing.

## Introduction

Deformational plagiocephaly (DP) is a multiplanar asymmetric and a non-synostotic skull deformity that can occur either before or after birth (1–3). It is considered the most common type of cranial asymmetry in infants (4). DP typically presents as flattening on one side of the occiput, but it can also affect both sides, as seen in brachycephaly. Reported cases have increased by up to 600% as reported in the literature after “Back to Sleep” (5). “Back to sleep” was a campaign launched in April 1992 by the American Academy of Pediatrics (AAP), was geared towards preventing sudden infant death syndrome (SIDS) (6). About 70% of normal infants in Japan, between 1 and 7 months-old, have DP (7).

The primary associated factors for developing DP are specific nursing habits, motor development, and positional preference (8). Other reported risk factors are premature birth, multiple births, restrictive intrauterine environment, delivery by forceps or vacuum extraction and male gender (3, 9, 10). DP is also often associated with torticollis which usually presents with the child holding their heads to one side due to limited head tilting or rotation (11–13). This leads to the child preference to sleep in one side than the other which in turn leads too DP.

DP results from prolonged external pressure on an infant's skull, particularly at points of surface contact, may inhibit localized cranial growth due to mechanical loading. As a compensatory response, cranial expansion is redirected toward regions experiencing lower compressive forces, frequently resulting in asymmetrical skull development (14–16).

DP is usually diagnosed by physical examination. Imaging modalities such as cranial ultrasound, Magnetic Resonance Imaging (MRI), or Computed Tomography (CT) scan may be used to exclude the presence of craniosynostosis. Various geometric measurements can be used to assess the severity of DP, with the Cranial Vault Asymmetry Index (CVAI) being the most important and commonly relied upon (17, 18).

DP may be treated through counter-positioning therapy, physical therapy, and cranial helmet therapy (CHT). Minor cranial deformity with DP can be treated by counter-positioning therapy, based on the guidelines from the Congress of Neurological Surgeons (CNS), the American Association of Neurological Surgeons (AANS), and the American Academy of Pediatrics (AAP) (19). Moderate DP can be treated using counter-positioning therapy and physical therapy (13, 20). For infants with severe DP, CHT is more effective than relying solely on the natural course of improvement (21, 22).

The effectiveness of CHT in treating DP depends on the infant's age (23). Molding CHT, often made from thermoplastic materials, was recommended to start with infants with severe and mild deformities at the age of 4 and 6 months-old, respectively (24). Plaster of Paris (PoP) is used to capture the skull geometries and produce a positive plaster mold and apply the required rectification; thereafter, thermoplastic material is drapped to have the molding Untreated, DP has been reported to influence cranial shape and potentially impact functional and developmental outcomes (25, 26). While 85% of the skull growth occurs within the first year of birth (17), this limits the window for early assessment and intervention using CHT (18).

While few studies have explored thermoplastic CHT (21), recent studies (27, 28), have demonstrated the clinical utility of 3D-printed CHT in both Japanese and European cohorts. Therefore, the aim of this study was to assess the effectiveness of 3D-printed CHT in treating infants with DP, in the middle east region.

## Methods

Ethical approval was obtained from the Ministry of Health (IRB-409). Medical records from a private medical center were reviewed to identify infants diagnosed with DP and treated using 3D-printed CHT. Rodin4D software (Rodin4D, Eqwal Group, France) was used to extract relevant data, including the infants' age, number of visits, number of helmets used, treatment duration, and CVAI scores.

### Data acquisition and measurement procedures

**Figure 1:**
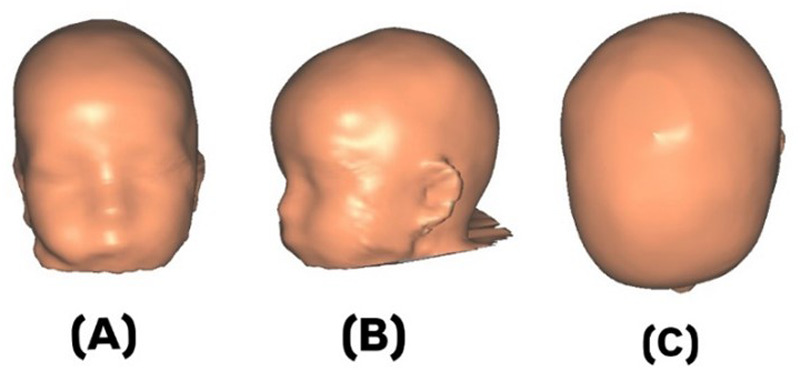
3D-images of an infant's skull with deformational plagiocephaly (DP): **(A)** anterior, **(B)** lateral, and **(C)** superior views.

This study involved a retrospective review of clinical records and 3D scan data from a private medical center. A structural sensor scanner with a resolution of 1,280 × 960 pixels, synchronized with Rodin4D software (Rodin4D, Eqwal Group, France), was used during treatment to capture the geometry of each infant's head and generate 3D images of the skull (Figure 1). During scanning, the infant's head was positioned neutrally, and anatomical landmarks—including the glabella, opisthocranion, ears, occipital bone, orbital area, exocanthion, and tragion were identified using reference patches placed on the scalp.

The CVAI was measured using diagonal measurements at 30°, following the method of Loveday and de Chalain (29), using Rodin4D software (Rodin4D, Eqwal Group, France). The severity of DP was classified according to the criteria established by Loveday and de Chalain (29). They classified CVAI < 3.5% as within the normal range (no significant asymmetry), CVAI between 3.5% and 6.25% as mild to moderate plagiocephaly, and CVAI ≥ 6.25% as severe plagiocephaly. Scan durations ranged from 3 to 5 min. The resulting 3D scans were cleaned and processed using Meshmixer software (Autodesk, United States).

### 3D-helmet design and data processing

**Figure 2:**
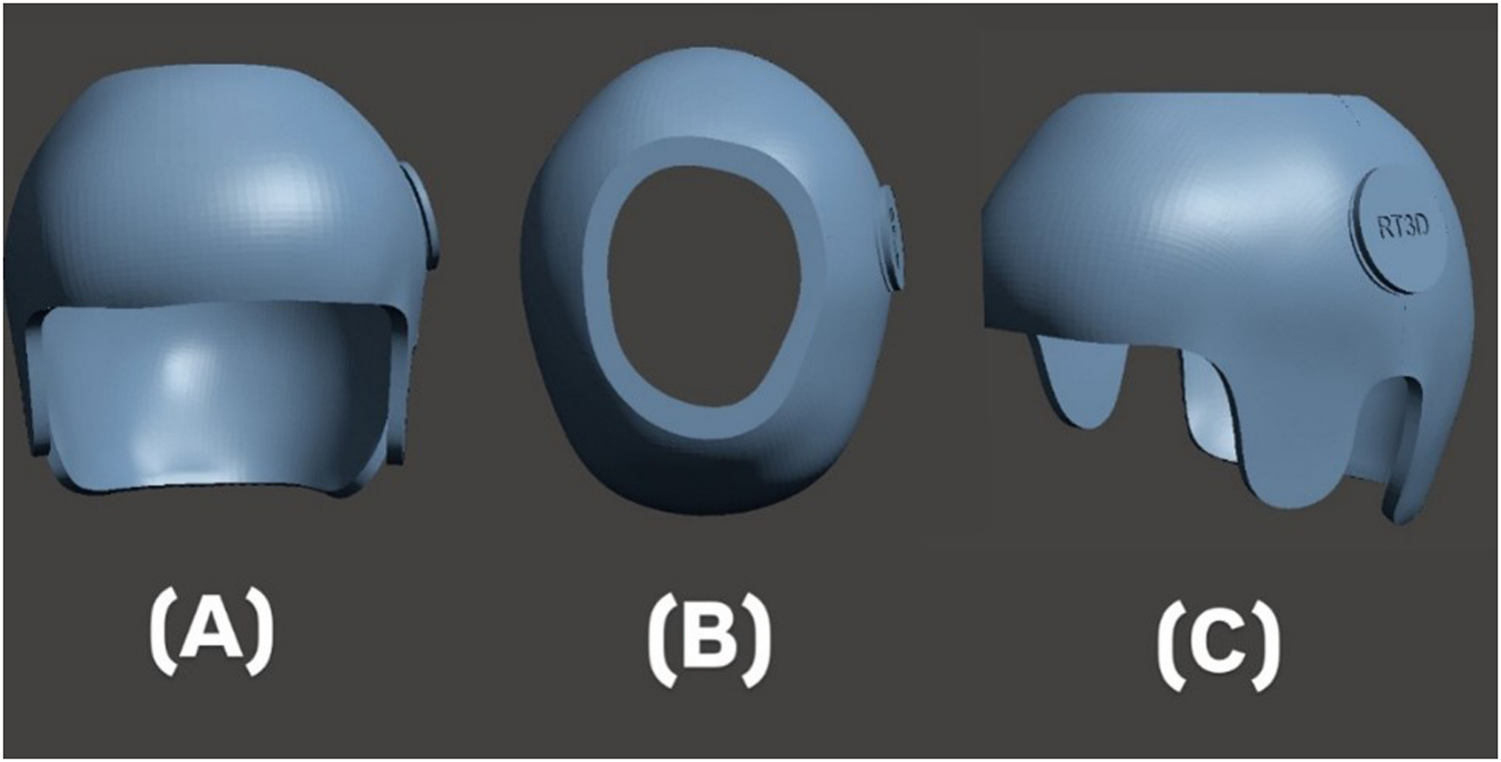
3D-design of the helmet: **(A)** anterior, **(B)** superior, and **(C)** lateral views.

As part of the retrospective review, digital records of helmet design workflows were analyzed. Rodin4D-Neo software (Rodin4D, Eqwal Group, France) was used to modify and rectify the scanned 3D skull models captured during clinical treatment. Meshmixer software (Autodesk, United States) was then used to define the helmet trim-line, which was extended from above the glabella to the inferior occipital bone and over vertebra C2. To further customize the design, ZBrush software (Maxon, Germany) was employed to convert the triangular mesh into a quad mesh, allowing for additional editing in CAD software. This process enabled the addition of personal design features such as surface patterns and text (Figure 2).

### 3D-helmet printing process

As part of the retrospective review, digital records of the 3D helmet printing process were analyzed. The helmet designs originally created using Fusion 360 software (Autodesk, United States). Each helmet weighed approximately 180–220 g. It included 4 mm Plastazote padding for comfort and ventilation holes to improve breathability (Figure 2). Ventilation holes were incorporated to reduce sweating, and Plastazote padding was affixed to the inner surface to absorb pressure and allow for adjustments during follow-up visits.

**Figure 3:**
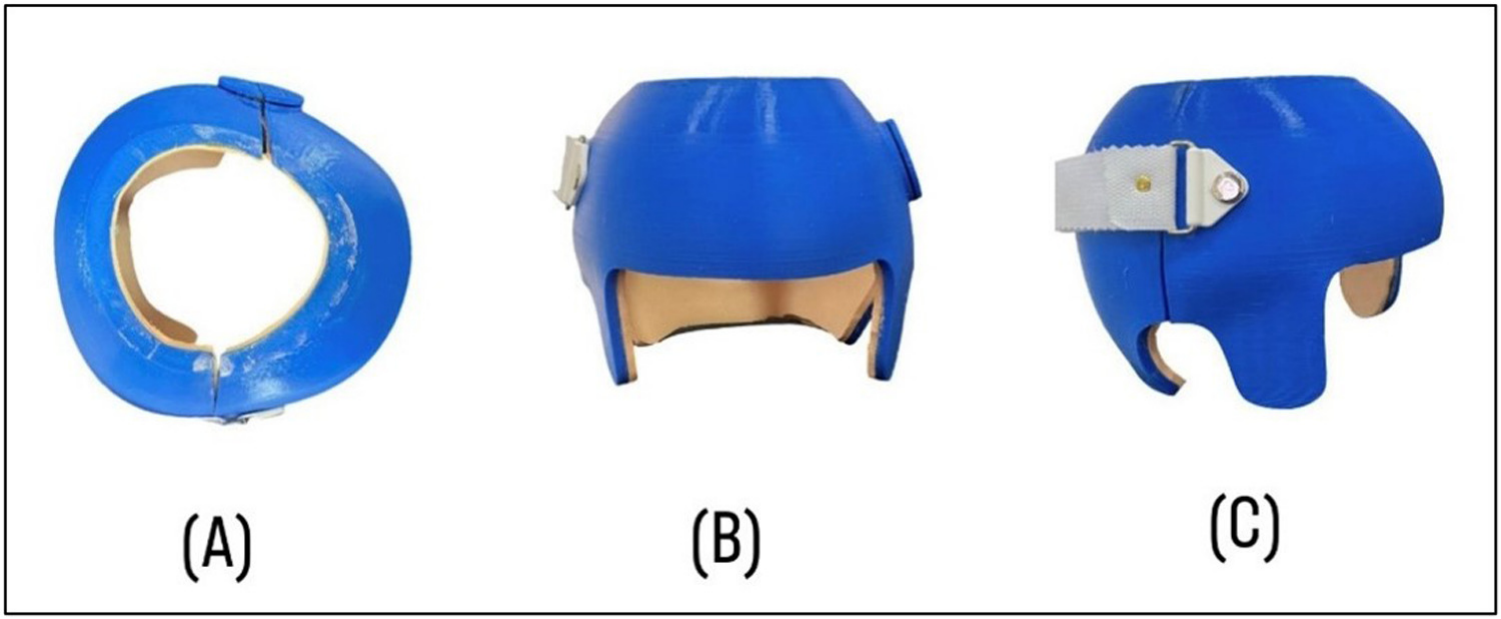
3D-constructed helmet used in this study: **(A)** superior, **(B)** anterior, and **(C)** lateral views.

The finalized 3D designs were sliced using Creality Print software (Creality 3D Technology Co. Ltd., Longhua District, Shenzhen, China), which generated the necessary printer instructions. A cube infill pattern was selected to provide optimal impact resistance, with print parameters adjusted to balance helmet rigidity and flexibility. The following printer settings were recorded: nozzle temperature 220°C, bed temperature 60°C, 60% infill using cube infill type, indirect extrusion, printing speed 180 mm/s, and nozzle size 0.4 mm. Helmets were fabricated using a Creality V3 Plus printer and PET-G (Polyethylene Terephthalate Glycol) filament (Figure 3).

### Helmet fitting

Clinical records were reviewed to assess the helmet fitting procedures followed during treatment. After the initial quality check of the 3D-printed helmet, clinicians evaluated helmet readiness and fit. Pressure points were inspected for signs of excessive pressure, friction, or skin irritation. A strap was attached to facilitate easy donning and doffing of the helmet. According to treatment documentation, the helmets were worn for approximately 23 h per day, with regular follow-ups to ensure fit and comfort.

### Follow-up and discharge

Follow-up appointments were scheduled every 3–4 weeks. To evaluate the effectiveness of the 3D-printed helmet, the CVAI was recalculated and compared with previous values. If the CVAI remained above 4, a new follow-up appointment was scheduled. Adjustments to the helmet were made using the Plastazote padding to improve fit and comfort. In cases where adjustment was insufficient or the infant's head had outgrown the helmet, new measurements were taken to design a replacement helmet. This ensured adaptability and minimized treatment interruptions, potentially leading to improved outcomes.

### Statistical analysis

Statistical analysis was conducted using IBM SPSS Statistics version 22. Descriptive statistics (mean ± SD) summarized infant characteristics and treatment data, including age, number of visits, number of helmets, treatment duration, and CVAI scores. To assess changes in CVAI, a Linear Mixed Model was applied with random intercepts for each participant. Fixed effects included time (pre- vs. post-treatment), gender, age, and treatment duration. The model was estimated using Restricted Maximum Likelihood (REML). A Wilcoxon signed-rank test was also used as a non-parametric confirmation of the CVAI change, given the small sample size. Statistical significance was defined as *p* < 0.05.

## Results

Eleven infants with moderate to severe DP were included in this study. The descriptive characteristics and treatment outcomes for the study participants are summarized in Table 1. Infants initiated 3D-printed helmet therapy at a mean age of 6.4 ± 2.7 months. The average number of clinical visits was 3.8 ± 0.9, with most infants requiring 1–2 helmets (mean = 1.5 ± 0.5) and undergoing approximately 1.5 ± 0.5 adjustments during treatment. The mean duration of therapy was 9.7 ± 1.7 weeks. No adverse events or complications, such as skin irritation or poor compliance, were reported in the clinical records reviewed.

**Table 1:** Descriptive statistics of infant characteristics and treatment outcome measures.

Variable	Range	Mean ± SD
Age (months)	3–12	6.4 ± 2.7
Number of clinical visits	3–5	3.8 ± 0.9
Number of helmets	1–2	1.5 ± 0.5
Number of adjustments	0–3	1.5 ± 0.5
Treatment duration (weeks)	9–14	9.7 ± 1.7
CVAI before treatment (%)	4–13	7.7 ± 2.8
CVAI after treatment (%)	1–4	3.1 ± 1.0

3D-printed CHT was associated with a statistically and clinically meaningful improvement in cranial symmetry. The CVAI significantly decreased from a pre-treatment mean of 7.7 ± 2.8% to a post-treatment mean of 3.1 ± 1.0% (*p* < 0.001). A Linear Mixed Model confirmed a significant effect of CVAI score (Estimate = 4.64, SE = 0.80, *t* = 5.79, *p* < 0.001), reflecting a consistent and substantial reduction in CVAI after helmet therapy (Table 2).

**Table 2:** Change in CVAI following 3D-printed CHT: linear mixed model results.

Effect	Estimate	Std. error	*t*-value	*p*-value
Intercept (baseline CVAI)	0.78	4.56	0.17	0.865
Gender (male vs. female)	−0.03	1.34	−0.02	0.983
Age at treatment initiation (months)	0.15	0.23	0.65	0.514
Treatment duration (weeks)	0.14	0.38	0.36	0.719
CVAI scores (pre-treatment vs. post- treatment)	4.64	0.80	5.79	**0** **.** **000**

Bold indicating statistically significant differences.

Other variables included in the model, gender (Estimate = −0.03, *p* = 0.983), age at the start of treatment (Estimate = 0.15, *p* = 0.514), and treatment duration (Estimate = 0.14, *p* = 0.719), were not statistically significant, indicating that the observed improvement was not dependent on these individual factors in this study (Table 2).

To account for the small sample size and validate the primary findings, a Wilcoxon signed-rank test was conducted. The test confirmed a statistically significant reduction in CVAI between baseline and follow-up (*Z* = −2.84, *p* = 0.004), supporting the result observed in the Linear Mixed Model.

## Discussion

Various treatment options for DP have been reported in the literature, with the most cited being active counter-positioning (ACP) and cranial helmet therapy (CHT). Loveday and de Chalain reported that ACP generally produced slightly better outcomes than CHT, possibly due to challenges associated with helmet manufacturing and fitting (29). However, other studies have found CHT to be more effective than ACP for treating DP (30, 31). CHT is typically indicated for moderate to severe cases (32–34). Additionally, ACP has been shown to require a significantly longer treatment duration—up to two to three times longer than CHT to achieve similar results (29, 33). Side effects associated with CHT are usually mild and can be effectively prevented or managed through proper parental guidance (35, 36). A combination of both ACP and CHT may offer the most effective approach for managing positional plagiocephaly (29). However, in this study only 3D-printed CHT was utilized.

The optimal starting age for CHT in infants with DP remains controversial in the literature. Studies reported that 6 months is the ideal age to initiate CHT (23, 35, 37). This is partly because some researchers argue that CHT is not recommended before 6 months, as rapid brain growth during this period can lead to significant spontaneous improvement (35). However, other studies recommend initiating CHT between 4 and 8 months of age (18, 20, 21, 24, 34, 38). Clarren et al. (35) reported that CHT is effective for treating DP, especially in severe cases when initiated before 6 months of age (37). Several studies have emphasized that an early start, before 4–6 months, is a critical factor in successful treatment, as it takes advantage of the rapid cranial growth during the first year of life (7, 18, 23, 39, 40). Han et al. concluded that initiating CHT after 6 months of age significantly reduced CVAI improvement rates and increased therapy duration (41). In contrast, Aihara et al. found that the starting age has only a small effect on treatment outcomes (2). In the United States, CHT is generally not recommended after the age of 2-years-old, due to the cessation of significant cranial growth (2). In Japan, CHT is generally not recommended after infancy (12 months), as per guidelines from the Japanese Society for Pediatric Neurosurgery, which emphasize limited effectiveness beyond this age (2). In our study, all treated infants were 12 months old or younger, which aligns with previous research. However, no significant correlation was found between age at treatment initiation and treatment outcomes. This may be due to the small sample size and the relatively narrow age range of the treated infants (Table 2).

Many studies have identified male gender as a risk factor for DP (26, 42, 43). Additionally, one study found that affected. But not really ales with a history of uterine constraint were at the highest risk for subsequent school problems (26). In our study, 8 boys and 3 girls were included. However, no significant correlation was found between gender and DP (Table 2), which may be due to the small sample size.

The duration of CHT can impact head circumference and symmetrical ratio. Studies have shown that cranial symmetry improves with longer treatment durations (2). For severe DP, a treatment duration of 8–12 weeks is typically recommended (21, 44, 45). This aligns with the findings of the present study, which reported a duration of 9–14 weeks (Table 1). The number of 3D-printed helmets fitted and the number of adjustments in this study ranged from 1 to 2 and 0–3, respectively, corresponding to the severity of DP in each infant (Table 1). However, no significant differences in cranial vault asymmetry index (CVAI) scores were observed based on treatment duration (Table 2), which may be attributed to the small sample size.

Children with a history of DP may continue to exhibit measurable cranial flattening and asymmetry up to 36 months of age if left untreated (46). Several studies have reported positive outcomes from CHT in treating DP (2, 13, 21, 22, 34, 47, 48). The CVAI was reduced from 7.7 ± 2.8% to 3.1 ± 1.0% (Table 1), indicating a shift from severe deformity to a minor level. This evidence demonstrates that 3D-printed CHT is effective in managing DP like the molding CHT. Furthermore, there were statistically significant differences in CVAI scores before and after fitting infants with the 3D-printed helmets (Table 2). This magnitude of change is consistent with prior research demonstrating the effectiveness of helmet therapy. Kluba et al. (23) reported a mean CVAI reduction of 3.5% using traditional molding helmets, while Noto et al. (21) found comparable improvements in a cohort of Japanese infants. Similarly, Kim et al. (49) reported a CVAI reduction of 4.3%, further supporting the therapeutic efficacy of helmet intervention for moderate to severe positional plagiocephaly. Cevik et al. (40) also documented a 3.5% decrease in CVAI, reinforcing the reliability of helmet therapy in achieving clinically significant cranial symmetry. Collectively, these findings validate our results and highlight the consistent impact of helmet therapy across different populations and clinical settings. Although the CVAI scores obtained using the 3D-printed and molded CHT were similar, the 3D-printed approach offers several advantages. It helps keep the infant clean during skull measurements, reduces fear and crying, and saves time and effort for practitioners. Additionally, it provides accurate digital data that can be easily compared during follow-ups. A new helmet can be quickly reprinted in case of loosening or damage, and a variety of designs and colors can be offered. Furthermore, digital measurements can be shared internationally for modification and printing.

In addition to prior studies, Cho et al. (50) investigated the effectiveness of helmet therapy across different cranial shapes and found that the morphological classification of skull deformities may impact treatment response, with asymmetrical types responding more favorably to helmet use. This insight suggests that individualized helmet designs, such as those enabled through 3D printing, could enhance therapeutic outcomes by better accommodating specific cranial geometries.

Similarly, Kim et al. (49) evaluated helmet therapy in infants with moderate to severe positional plagiocephaly and concluded that it led to significant CVAI improvements, reinforcing the role of helmeting as a primary intervention. Their findings align with our results, particularly in demonstrating that CVAI reductions are substantial and clinically meaningful even with a modest treatment duration, further validating the applicability of 3D-printed helmets.

Van Cruchten and Feijen (51) conducted a 5-year follow-up study, emphasizing the sustained benefits of helmet therapy over time. Their long-term perspective underscores the importance of early and effective treatment to ensure lasting correction of cranial asymmetry. These results support our current outcomes and highlight the potential for 3D-printed helmet therapy to achieve similarly enduring results, especially given its precision and adaptability.

Finally, it is worth emphasizing that 3D printing offers a substantial cost advantage. The average cost of a 3D-printed helmet in our study was approximately $150 USD, in contrast to traditional molded helmets, which often range between $300 and 500 USD. In resource-limited settings or national health systems where cost-effectiveness is crucial, this reduction in material and labor costs could facilitate broader access to cranial orthoses. Moreover, 3D printing allows for rapid prototyping, quick reprints in case of fit issues, and personalized cosmetic features—all without compromising clinical effectiveness.

## Conclusion

This study demonstrated that CHT, including those produced using 3D printing, effectively reduced cranial asymmetry in infants with mild to severe DP. Initiating treatment before 6 months of age was associated with more favourable outcomes. The reduction in asymmetry, as measured by the CVAI, was statistically significant and consistent across age, gender, and duration of helmet use, underscoring the broad clinical applicability of CHT. While these findings support the use of CHT in managing DP, the specific contribution of the 3D-printing method to treatment outcomes remains unclear. Further research involving larger, more diverse cohorts is warranted to confirm these results and explore whether manufacturing methods influence clinical effectiveness or offer other practical advantages, such as customization or cost-efficiency.

## Study limitations

This study is limited by its small sample size (*n* = 11) and retrospective design, which restricts the generalizability of the findings and precludes a detailed cost-effectiveness analysis. The sample size was constrained by the fact that only one center in Jordan currently provides 3D-printed cranial helmet therapy (CHT), limiting the ability to assess the effects of multiple variables. Additionally, a longitudinal follow-up may be valuable to evaluate the long-term effects of deformational plagiocephaly (DP) on head shape as children grow.

Nevertheless, the use of 3d printing presents a promising avenue for scalable and individualized cranial orthoses, a detailed cost analysis per patient was not feasible due to the small cohort size. Future research should include cost-effectiveness assessments, especially when considering broader implementation in low-resource settings.

## Data Availability

The original contributions presented in the study are included in the article/Supplementary Material, further inquiries can be directed to the corresponding author.
